# Supplementary material for: Similarities and differences in the microbial structure of surface soils of different vegetation types
Source: PeerJ. 2023 Oct 19;11:e16260. doi: 10.7717/peerj.16260 (PMC10590577; doi:10.7717/peerj.16260)
Supplement: Supplemental Information 1 — C/N: carbon to nitrogen ratio; N/P: nitrogen to phosphorus ratio; C/P: carbon to phosphorus ratio; HH: a woodland with the dominant tree species Horsfieldia hainanensis; DP: a woodland with the dominant tree species Drypetes perreticulata; ZM: a Zea mays farmland; CR: a Citrus reticulata farmland. [file peerj-11-16260-s001.docx]

**Table 1- raw data.** Soil chemical properties of different vegetation types

|  | **pH** | **Total carbon/g kg^-1^** | **Total nitrogen/g kg^-1^** | **Total phosphorus/g kg^-1^** | **C/N** | **N/P** | **C/P** |
| --- | --- | --- | --- | --- | --- | --- | --- |
| HH1 | 6.51 | 71.62 | 6.32 | 0.36 | 11.33 | 17.33 | 196.35 |
| HH2 | 6.58 | 68.55 | 6.34 | 0.38 | 10.81 | 16.83 | 181.92 |
| HH3 | 6.18 | 72.22 | 6.48 | 0.33 | 11.15 | 19.64 | 218.85 |
| HH4 | 6.67 | 68.48 | 6.21 | 0.31 | 11.03 | 19.77 | 217.98 |
| DP1 | 6.91 | 208.92 | 13.74 | 0.47 | 15.21 | 29.51 | 448.73 |
| DP2 | 6.87 | 215.93 | 14.00 | 0.39 | 15.42 | 36.17 | 557.91 |
| DP3 | 6.94 | 199.51 | 12.90 | 0.41 | 15.47 | 31.46 | 486.53 |
| DP4 | 6.92 | 211.23 | 14.10 | 0.38 | 14.98 | 37.34 | 559.34 |
| CR1 | 6.26 | 20.21 | 2.28 | 0.37 | 8.86 | 6.22 | 55.13 |
| CR2 | 6.27 | 20.87 | 2.32 | 0.34 | 9.00 | 6.79 | 61.10 |
| CR3 | 6.29 | 20.83 | 2.32 | 0.34 | 8.98 | 6.86 | 61.60 |
| CR4 | 6.23 | 20.48 | 2.28 | 0.36 | 8.98 | 6.28 | 56.44 |
| ZM1 | 5.82 | 18.58 | 1.88 | 0.50 | 9.88 | 3.79 | 37.41 |
| ZM2 | 6.40 | 17.76 | 1.79 | 0.43 | 9.92 | 4.15 | 41.17 |
| ZM3 | 6.26 | 17.65 | 1.76 | 0.42 | 10.03 | 4.17 | 41.78 |
| ZM4 | 6.28 | 17.97 | 1.79 | 0.56 | 10.04 | 3.17 | 31.85 |

C/N: carbon to nitrogen ratio; N/P: nitrogen to phosphorus ratio; C/P: carbon to phosphorus ratio; HH: a woodland with the dominant tree species *Horsfieldia hainanensis*; DP: a woodland with the dominant tree species *Drypetes perreticulata*; ZM: a *Zea mays* farmland; CR: a *Citrus reticulata* farmland.
