# Supplementary material for: Similarities and differences in the microbial structure of surface soils of different vegetation types
Source: PeerJ. 2023 Oct 19;11:e16260. doi: 10.7717/peerj.16260 (PMC10590577; doi:10.7717/peerj.16260)
Supplement: Table S1 — Notes: The values in the table indicate the p-value of the corresponding two cultivars in that index. HH: a woodland with the dominant tree species Horsfieldia hainanensis; DP: a woodland with the dominant tree species Drypetes perreticulata; ZM: a Zea mays farmland; CR: a Citrus reticulata farmland. [file peerj-11-16260-s002.docx]

**Table S1** Alpha diversity index p-value post hoc test table

| **Bacterium** | | | | **Fungus** | | | |
| --- | --- | --- | --- | --- | --- | --- | --- |
| **Chao 1 index** | | | | | | | |
| Group | HH | DP | ZM | Group | HH | DP | ZM |
| DP | 0.83 | NaN | NaN | DP | 0.55 | NaN | NaN |
| ZM | 0.48 | 0.83 | NaN | ZM | 0.15 | 0.41 | NaN |
| CR | 0.014 | 0.11 | 0.48 | CR | 0.0065 | 0.038 | 0.47 |
| **Shannon index** | | | | | | | |
| Group | HH | DP | ZM | Group | HH | DP | ZM |
| DP | 0.36 | NaN | NaN | DP | 0.36 | NaN | NaN |
| ZM | 0.36 | 0.88 | NaN | ZM | 0.22 | 0.66 | NaN |
| CR | 0.0065 | 0.35 | 0.32 | CR | 0.0065 | 0.35 | 0.41 |
| **Pielous index** | | | | | | | |
| Group | HH | DP | ZM | Group | HH | DP | ZM |
| DP | 0.32 | NaN | NaN | DP | 0.94 | NaN | NaN |
| ZM | 0.35 | 0.82 | NaN | ZM | 1 | 0.38 | NaN |
| CR | 0.005 | 0.35 | 0.35 | CR | 1 | 0.79 | 1 |

Notes:

The values in the table indicate the p-value of the corresponding two cultivars in that index.

HH: a woodland with the dominant tree species *Horsfieldia hainanensis*; DP: a woodland with the dominant tree species *Drypetes perreticulata*; ZM: a *Zea mays* farmland; CR: a *Citrus reticulata* farmland.
