# Supplementary material for: Similarities and differences in the microbial structure of surface soils of different vegetation types
Source: PeerJ. 2023 Oct 19;11:e16260. doi: 10.7717/peerj.16260 (PMC10590577; doi:10.7717/peerj.16260)
Supplement: Table S2 — Notes: HH: a woodland with the dominant tree species Horsfieldia hainanensis; DP: a woodland with the dominant tree species Drypetes perreticulata; ZM: a Zea mays farmland; CR: a Citrus reticulata farmland. [file peerj-11-16260-s003.docx]

**Table S1** Analysis of the composition of soil microbial community composition of different vegetation types at different levels of taxonomic units

| **Taxon** | | | **HH** | **DP** | **ZM** | **CR** |
| --- | --- | --- | --- | --- | --- | --- |
| Bacterium | Phylum | Actinobacteria | 24.81% | 36.98% | 42.15% | 42.99% |
|  |  | Proteobacteria | 36.31% | 34.70% | 19.47% | 17.39% |
|  |  | Acidobacteria | 15.38% | 14.57% | 9.63% | 11.16% |
|  |  | Chloroflexi | 5.45% | 3.28% | 15.42% | 18.57% |
|  |  | Gemmatimonadetes | 1.79% | 1.53% | 5.03% | 3.18% |
|  |  | Rokubacteria | 4.50% | 2.95% | 1.63% | 1.40% |
|  |  | Planctomycetes | 2.32% | 1.88% | 0.94% | 0.96% |
|  |  | Bacteroidetes | 2.41% | 0.87% | 0.54% | 0.34% |
|  | Genus | Subgroup_6 | 8.92% | 7.18% | 2.84% | 1.97% |
|  |  | AD3 | 0.00% | 0.01% | 5.11% | 7.62% |
|  |  | Acidothermus | 0.48% | 0.01% | 4.52% | 7.70% |
|  |  | 67-14 | 1.71% | 7.25% | 1.88% | 1.42% |
|  |  | Rokubacteriales | 4.44% | 2.95% | 1.63% | 1.40% |
|  |  | bacteriap25 | 4.65% | 3.91% | 0.68% | 0.65% |
|  |  | Gaiella | 1.90% | 0.98% | 1.71% | 1.61% |
|  |  | Rubrobacter | 0.82% | 1.75% | 1.33% | 2.16% |
|  |  | Solirubrobacter | 0.94% | 2.30% | 1.73% | 0.87% |
|  |  | JG30-KF-AS9 | 0.00% | 0.01% | 2.90% | 2.87% |
|  |  | IMCC26256 | 1.15% | 0.99% | 1.69% | 1.44% |
|  |  | TK10 | 0.38% | 0.69% | 1.75% | 2.06% |
|  |  | Mycobacterium | 0.59% | 2.25% | 1.13% | 0.89% |
|  |  | Bradyrhizobium | 0.85% | 1.15% | 1.43% | 0.90% |
|  |  | RB41 | 0.77% | 1.41% | 0.89% | 1.23% |
| Fungus | Phylum | Ascomycota | 73.82% | 55.17% | 74.45% | 85.46% |
|  |  | Basidiomycota | 13.92% | 31.91% | 24.10% | 12.44% |
|  |  | Mortierellomycota | 3.86% | 0.02% | 0.43% | 0.19% |
|  | Genus | Fusarium | 4.15% | 2.00% | 10.51% | 17.84% |
|  |  | Aspergillus | 5.89% | 3.34% | 5.38% | 3.40% |
|  |  | Hygrocybe | 0.00% | 17.03% | 0.00% | 0.00% |
|  |  | Penicillium | 1.56% | 1.17% | 1.29% | 6.90% |
|  |  | Humicola | 4.58% | 0.64% | 1.76% | 3.69% |
|  |  | Talaromyces | 1.20% | 0.60% | 4.26% | 3.41% |
|  |  | Staphylotrichum | 3.70% | 0.07% | 2.65% | 1.80% |
|  |  | Paramyrothecium | 0.01% | 0.00% | 0.00% | 7.25% |
|  |  | Basidioascus | 0.02% | 0.02% | 0.60% | 5.26% |

Notes:

HH: a woodland with the dominant tree species *Horsfieldia hainanensis*; DP: a woodland with the dominant tree species *Drypetes perreticulata*; ZM: a *Zea mays* farmland; CR: a *Citrus reticulata* farmland.
